# Gene-Edited Stem Cells for Ischemic Vascular Disease: Current Advances and Future Perspectives

**DOI:** 10.3390/cimb48070681

**Published:** 2026-07-02

**Authors:** Seongho Han, Sung-Whan Kim

**Affiliations:** 1 Department of Family Medicine, College of Medicine, Dong-A University, Busan 49201, Republic of Korea; 2 Department of Medicine, College of Medicine, Catholic Kwandong University, Gangneung 25601, Republic of Korea; 3 International St. Mary’s Hospital, Incheon 22711, Republic of Korea

**Keywords:** genome editing, CRISPR/Cas9, stem cell engineering, ischemic vascular disease, vascular regeneration

## Abstract

Ischemic vascular diseases remain a leading cause of morbidity and mortality worldwide and are frequently associated with irreversible tissue damage. Although stem cell-based therapies have shown promise for vascular regeneration, their clinical translation has been limited by poor survival, insufficient engraftment, functional heterogeneity, and immune rejection. Recent advances in genome-editing technologies, including CRISPR/Cas9, base editing, and prime editing, have provided powerful tools for overcoming these limitations through precise genetic modification of stem cells. Gene editing can enhance angiogenic potential, improve resistance to ischemic stress, augment paracrine activity, promote endothelial maturation, and reduce immunogenicity. In this review, we outline the current genome-editing toolbox and its application to stem cell engineering for vascular regeneration in ischemic disease. We also examine emerging therapeutic concepts, universal donor cell platforms, and key issues in safety and ethics, with a focus on translational pathways. Taken together, advances at the interface of genome editing and stem cell biology are likely to accelerate the development of regenerative therapies that deliver more durable vascular repair in ischemic vascular disease.

## 1. Introduction

Ischemic vascular diseases, including peripheral artery disease, myocardial infarction, and ischemic stroke, remain among the leading causes of morbidity and mortality worldwide [1]. Although substantial advances in pharmacological therapies and revascularization procedures have improved clinical outcomes, many patients continue to suffer from progressive tissue ischemia and irreversible organ damage [2]. Current treatment strategies primarily aim to restore blood flow and prevent disease progression; however, they generally do not regenerate damaged vascular networks or fully reverse established tissue injury [3]. Therefore, the development of regenerative approaches capable of promoting vascular repair and functional tissue recovery remains an important unmet clinical need.

Stem cell-based therapies have emerged as promising candidates for ischemic tissue regeneration because of their ability to stimulate angiogenesis, modulate inflammatory responses, and support endogenous repair processes [4]. Mesenchymal stem cells (MSCs), endothelial progenitor cells (EPCs), and induced pluripotent stem cell (iPSC)-derived vascular cells have shown encouraging therapeutic effects in numerous preclinical studies [4]. Nevertheless, clinical translation has been hindered by several biological limitations, including poor cell survival after transplantation, limited engraftment efficiency, inadequate functional integration, and heterogeneity among cell products [5]. These challenges have contributed to inconsistent therapeutic outcomes and have restricted the widespread clinical adoption of stem cell-based therapies for ischemic vascular diseases.

Recent advances in genome-editing technologies have created new opportunities to overcome these limitations. The development of CRISPR/Cas9 and next-generation precision-editing platforms, including base editing and prime editing, enables targeted modification of genes involved in angiogenesis, cell survival, stress adaptation, and immune regulation [6]. Through genetic engineering, stem cells can be optimized to enhance regenerative potency, improve resistance to hostile ischemic microenvironments, and reduce immune-mediated rejection after transplantation [7]. Recent progress in both stem cell biology and genome editing has made their combination a promising strategy for next-generation regenerative therapies for ischemic vascular diseases. In this review, we summarize recent advances in genome-editing technologies and discuss how these tools can be applied to enhance stem cell-mediated vascular regeneration and tissue repair.

## 2. Advances and Characteristics of Gene-Editing Technologies

**Figure 1:**
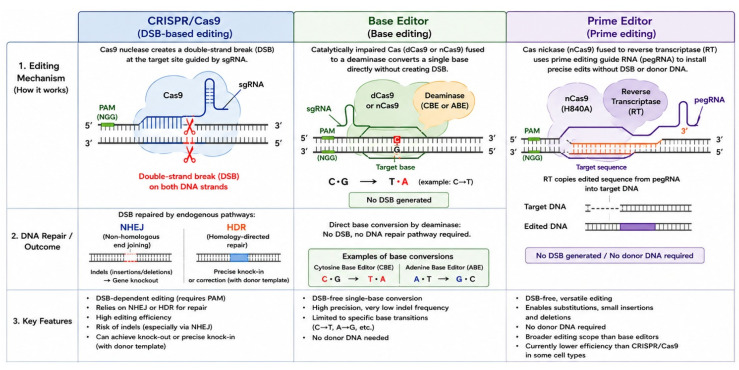
Overview of major gene-editing platforms and their characteristics. This figure compares the three principal genome-editing technologies currently used in regenerative medicine and stem cell engineering: CRISPR/Cas9, Base Editors (BEs), and Prime Editors (PEs). CRISPR/Cas9-mediated genome editing: CRISPR/Cas9 introduces a site-specific double-strand break (DSB) directed by a single-guide RNA (sgRNA). Cellular repair through non-homologous end joining (NHEJ) typically generates insertions or deletions, enabling efficient gene knockout, whereas homology-directed repair (HDR) can facilitate precise gene knock-in. The key message of this panel is that CRISPR/Cas9 provides a versatile and highly efficient platform for gene disruption and targeted genome modification but remains dependent on DSB-mediated DNA repair. Base editing: Base editors combine a catalytically impaired Cas protein with a nucleotide deaminase to directly convert one DNA base into another without generating DSBs. Cytosine base editors (CBEs) mediate C-to-T conversions, whereas adenine base editors (ABEs) enable A-to-G conversions. The key message of this panel is that base editing allows precise correction of single-nucleotide variants while reducing genomic disruption and unwanted indel formation. Prime editing: Prime editors consist of a Cas9 nickase fused to a reverse transcriptase and are guided by a prime-editing guide RNA (pegRNA). This system enables precise nucleotide substitutions, insertions, and deletions without requiring DSBs or donor DNA templates. The key message of this panel is that prime editing offers the greatest editing versatility and precision, allowing a broad range of targeted genomic modifications with improved safety profiles. Abbreviations: Cas9, CRISPR-associated protein 9; DSB, double-strand break; sgRNA, single-guide RNA; PAM, protospacer adjacent motif; NHEJ, non-homologous end joining; HDR, homology-directed repair; dCas9, catalytically dead Cas9; nCas9, Cas9 nickase; CBE, cytosine base editor; ABE, adenine base editor; RT, reverse transcriptase; pegRNA, prime-editing guide RNA. The figure was generated using OpenAI ChatGPT (GPT-5.5) image generation tools and subsequently reviewed and edited by the authors.

Genome-editing technology has evolved rapidly over the past decade, moving well beyond the first generation of programmable nucleases. The modern genome-engineering toolbox now encompasses CRISPR/Cas9, base editors, prime editors, and a growing collection of high-fidelity Cas variants (Figure 1). Each platform operates through a distinct molecular mechanism and offers unique advantages, limitations, and therapeutic opportunities [8].

The emergence of precision-editing technologies has also shifted the field conceptually. Earlier approaches focused largely on gene disruption through the introduction of double-strand DNA breaks using CRISPR/Cas9 systems [9]. More recent platforms emphasize targeted correction and fine-tuning of gene function while minimizing collateral genomic damage through technologies such as base editing and prime editing [10]. In parallel, the development of high-fidelity Cas variants has improved editing specificity and reduced off-target activity, thereby enhancing the safety profile of genome engineering approaches [11]. This shift reflects a broader move in therapeutic genome engineering toward methods that balance efficacy with safety [8].

### 2.1. Mechanism of Action of CRISPR/Cas9

CRISPR/Cas9 remains the most widely used genome-editing platform and serves as the foundation for many subsequent editing technologies. Originally derived from a bacterial adaptive immune system, the platform employs a single-guide RNA to direct the Cas9 nuclease to a complementary DNA sequence located adjacent to a protospacer-adjacent motif (PAM) [12]. Once target recognition occurs, Cas9 introduces a double-strand break at the specified genomic locus [9].

Cellular DNA repair mechanisms then determine the final editing outcome. Repair through non-homologous end joining (NHEJ) often results in insertions or deletions that disrupt gene function, making this pathway particularly useful for gene knockout applications [8]. Alternatively, homology-directed repair (HDR) can be exploited to introduce precise sequence modifications when an appropriate donor template is provided [9].

One of the major strengths of CRISPR/Cas9 lies in its simplicity. Retargeting the system generally requires only modification of the guide RNA sequence, allowing rapid adaptation to new genomic targets. This flexibility has contributed to its widespread adoption in disease modeling, functional genomics, and stem cell engineering [9].

Despite these advantages, several limitations remain. Dependence on double-strand DNA breaks introduces the possibility of unintended repair outcomes, including indel formation and chromosomal rearrangements. Furthermore, editing efficiency may vary across cell types, and off-target cleavage remains a concern, particularly in therapeutic applications where long-term genomic stability is essential [11]. These challenges have motivated the development of newer editing platforms, including base editing and prime editing, which seek to retain programmability while minimizing genomic disruption [13].

### 2.2. High-Efficiency and High-Precision Gene-Editing Technologies

Although CRISPR/Cas9 has transformed genome engineering, concerns regarding double-strand break–associated toxicity and unintended genomic alterations have stimulated the development of alternative editing platforms. Among these, base editing and prime editing represent major advances toward more precise genome manipulation [10].

Base editors enable direct, programmable single-base conversions (e.g., C → T or A → G) without introducing double-strand DNA breaks. By coupling a catalytically impaired Cas protein with a nucleotide deaminase, these systems facilitate targeted base substitutions while minimizing the risk of insertions, deletions, and large-scale chromosomal rearrangements [14]. This capability is particularly valuable for correcting disease-causing point mutations, which account for a substantial proportion of inherited genetic disorders.

Prime editing extends this concept even further. Rather than relying on donor DNA templates or double-strand break repair, prime editors utilize a Cas9 nickase fused to a reverse transcriptase, guided by a prime editing guide RNA (pegRNA) that encodes the desired genetic change [13]. As a result, a wide variety of sequence modifications—including substitutions, insertions, and deletions—can be introduced with remarkable precision [13].

Alongside these innovations, extensive efforts have been directed toward engineering high-fidelity Cas9 variants. These engineered nucleases exhibit improved discrimination between intended and unintended genomic targets, thereby reducing off-target activity while maintaining robust on-target editing efficiency [15]. Such improvements are particularly important in stem cell applications, where rare genomic alterations may be clonally expanded during long-term culture and large-scale expansion.

Despite their advantages, next-generation genome editing platforms are not without limitations. Editing efficiency can vary substantially depending on both cell type and genomic locus [16]. In addition, protospacer adjacent motif (PAM) requirements and delivery constraints remain critical practical considerations [16]. The relatively large size of certain editor constructs further complicates viral vector-mediated delivery, particularly in adeno-associated virus (AAV)-based systems [16]. Consequently, further optimization will be required before these technologies achieve their full therapeutic potential.

### 2.3. Strategies for Minimizing Off-Target Effects

As genome-editing technologies move closer to clinical application, off-target activity has become one of the most intensively studied safety concerns. Unintended DNA modifications may compromise genomic stability and potentially affect therapeutic outcomes. For stem cell-based products, where edited cells may persist for extended periods after transplantation, ensuring genomic accuracy is particularly important [9].

Modern approaches to off-target reduction rely on multiple complementary strategies. Computational tools are now routinely used to design highly specific guide RNAs before experimental validation [9]. In parallel, engineered Cas variants with enhanced target specificity have substantially reduced unwanted cleavage events [17]. Additional improvements have been achieved through the use of chemically modified guide RNAs, paired nickase systems, and transient delivery approaches [16]. Rather than maintaining prolonged intracellular expression, many investigators now favor ribonucleoprotein complexes or mRNA-based delivery methods that limit the duration of editor activity. Restricting the editing window can significantly reduce opportunities for off-target interactions [16]. Advances in sequencing technologies have also improved the ability to detect rare editing events. Genome-wide profiling methods are increasingly incorporated into preclinical development pipelines, providing a more comprehensive assessment of editing specificity than was previously possible [9].

Even so, complete elimination of off-target effects remains difficult. Rare genomic alterations may escape detection during short-term studies, and their biological significance can be difficult to predict. Consequently, long-term genomic surveillance remains an essential component of therapeutic development [16].

## 3. Gene Editing-Based Stem Cell Engineering Strategies

Stem cell engineering has emerged as one of the most promising applications of genome-editing technology. By combining the self-renewal capacity of stem cells with programmable genetic modification, researchers can design cell products with properties that extend far beyond those of their unmodified counterparts [9].

In regenerative medicine, this convergence offers opportunities not only to correct disease-causing mutations but also to enhance cellular function in ways that improve therapeutic performance [18]. For ischemic vascular diseases, such modifications may influence angiogenesis, tissue integration, inflammatory responses, metabolic adaptation, and long-term survival following transplantation [18].

The scope of stem cell engineering has expanded considerably over the past decade. Early studies focused primarily on gene knockout and knock-in approaches [19]. More recently, advances in base editing, prime editing, CRISPR interference, CRISPR activation, and epigenetic editing have enabled increasingly sophisticated control of cellular behavior [20]. Importantly, some of these methods allow gene regulation without permanently altering the underlying DNA sequence [20].

Pluripotent stem cells have become particularly valuable in this context. Genome editing enables the generation of isogenic disease models in which specific genetic variants can be introduced, corrected, or modified within an otherwise identical cellular background [19]. Such systems provide powerful tools for investigating disease mechanisms and evaluating therapeutic interventions. Moreover, insights gained from these models frequently inform the development of clinically relevant cell products [18].

Beyond disease correction, considerable attention has shifted toward functional enhancement. In mesenchymal stromal cells and vascular progenitors, genome engineering is increasingly being used to strengthen resistance to oxidative stress, improve survival under ischemic conditions, and enhance the secretion of regenerative factors [18]. These modifications are intended to address the biological challenges that commonly limit therapeutic efficacy after transplantation.

Another major area of interest involves immune compatibility. The development of hypoimmunogenic stem cell products has the potential to overcome one of the principal barriers to allogeneic therapy [21]. Through targeted modification of antigen-presentation pathways and the introduction of immune-regulatory mechanisms, researchers are exploring strategies that may reduce immune rejection while preserving essential immune surveillance [21]. A particularly intriguing direction involves transcriptional and epigenetic reprogramming. Instead of permanently changing DNA sequences, these approaches seek to reshape cellular states by modulating gene-expression networks [20]. Such flexibility may prove advantageous when transient or reversible alterations are preferred.

Overall, these advances show that genome editing has moved beyond simple gene disruption and now functions as a versatile platform for sophisticated cellular engineering. As the field continues to mature, the integration of genetic, transcriptional, and epigenetic control is expected to play an increasingly important role in the development of next-generation regenerative therapies.

## 4. Gene Editing-Based Strategies for Stem Cell Differentiation and Functional Enhancement in Vascular Regeneration

**Figure 2:**
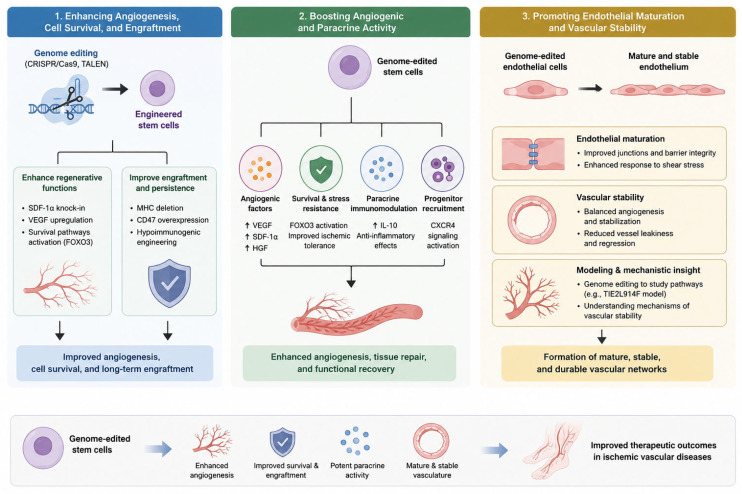
Genome-editing strategies to enhance the regenerative potential of stem cells for vascular regeneration. Schematic illustration of the major mechanisms by which genome editing improves stem cell-mediated vascular regeneration. Genome-editing technologies, including CRISPR/Cas9 and TALEN, can enhance therapeutic efficacy through multiple complementary approaches. First, genetic modifications such as SDF-1α knock-in, VEGF upregulation, and FOXO3 activation promote angiogenesis, improve ischemic tolerance, and increase cell survival and engraftment after transplantation. Second, genome editing enhances paracrine activity by stimulating the secretion of pro-angiogenic and immunomodulatory factors, including VEGF, hepatocyte growth factor (HGF), and interleukin-10 (IL-10), thereby facilitating tissue repair and functional recovery. Third, genome editing contributes to endothelial maturation and vascular stabilization by improving endothelial junction integrity, barrier function, mechanotransduction, and responses to shear stress. In addition, disease-modeling studies using edited endothelial cells have provided mechanistic insights into pathways regulating vascular stability. Collectively, these approaches promote the formation of mature and durable vascular networks, ultimately improving therapeutic outcomes in ischemic vascular diseases. Different panel colors denote separate mechanistic themes, and arrows indicate the progression from genome editing to engineered stem cells and ultimately to improved vascular regeneration. The figure was generated using OpenAI ChatGPT (GPT-5.5) image generation tools and subsequently reviewed and edited by the authors.

The therapeutic value of stem cells in vascular regeneration depends not only on their ability to differentiate into vascular cell types but also on their capacity to survive, integrate, and function within a hostile ischemic environment. Consequently, recent efforts have increasingly focused on using genome-editing technologies to optimize multiple aspects of stem cell performance simultaneously [9]. Rather than serving solely as tools for genetic correction, modern editing platforms are now being used to enhance lineage commitment, improve stress tolerance, strengthen paracrine activity, and promote long-term vascular stability [19]. These complementary strategies collectively aim to generate more effective and clinically relevant cell products for ischemic tissue repair (Figure 2).

### 4.1. Enhancing Angiogenesis, Cell Survival, and Engraftment

The restoration of functional vasculature remains a central objective of regenerative therapies for ischemic vascular diseases. Although stem cells possess intrinsic angiogenic and reparative properties, their therapeutic efficacy is often limited by inadequate neovascularization, poor survival within ischemic tissues, and insufficient long-term engraftment. Recent advances in genome-editing technologies have provided new opportunities to overcome these limitations through precise genetic modification of stem cells. Compared with conventional gene-transfer approaches, genome editing enables targeted and stable genomic modification, thereby improving therapeutic consistency and reducing the risks associated with random transgene integration [22].

One representative example is the TALEN-mediated knock-in of stromal cell-derived factor-1α (SDF-1α) into human amniotic mesenchymal stem cells. Zhang et al. demonstrated that SDF-1-edited cells exhibited enhanced angiogenic activity and significantly improved blood perfusion recovery, capillary formation, and limb salvage in a murine hindlimb ischemia model [23]. These results suggest that targeted genome engineering can improve the pro-angiogenic properties of stem cells and promote vascular regeneration in ischemic tissues. As shown in Table 1, this approach exemplifies how precise tuning of endogenous regenerative pathways can increase the therapeutic potential of stem cell-based interventions.

Beyond promoting angiogenesis, genome editing has also emerged as a promising approach for improving long-term graft persistence after transplantation. One of the major barriers to successful cell therapy is immune-mediated rejection, which contributes to progressive loss of transplanted cells and limits therapeutic durability. Recent studies have demonstrated that CRISPR/Cas9-mediated deletion of major histocompatibility complex (MHC)-related genes, combined with immune-evasive modifications such as CD47 overexpression, can generate hypoimmunogenic pluripotent stem cells with substantially reduced immunogenicity [21,27]. Deuse et al. reported that engineered pluripotent stem cells lacking key immune recognition molecules exhibited prolonged survival following transplantation, whereas Han et al. further demonstrated that multiplex genome editing could generate universal donor-like human pluripotent stem cells with enhanced graft persistence [21,27]. Although these studies were not performed specifically in ischemic vascular disease models, they provide important evidence that precise genome engineering can improve cell survival and transplantation outcomes.

Overall, current studies indicate that genome editing can improve stem cell-based regenerative therapies in two ways: by enhancing angiogenic and reparative functions and by supporting long-term persistence of transplanted cells. As genome-editing technologies continue to evolve, the integration of pro-regenerative genetic modifications with immune-evasive engineering strategies may facilitate the development of next-generation stem cell therapies for ischemic vascular diseases.

### 4.2. Boosting Angiogenic and Paracrine Activity

One representative strategy involves the targeted enhancement of vascular endothelial growth factor (VEGF) signaling. Cho et al. utilized targeted genome engineering to generate human umbilical cord blood-derived mesenchymal stem cells with controlled VEGF expression [25]. The engineered cells exhibited enhanced angiogenic activity, improved endothelial cell recruitment, and increased vascular network formation, suggesting that precise regulation of VEGF production can significantly augment the regenerative potential of stem cell therapies in ischemic tissues [25].

The stromal cell-derived factor-1 (SDF-1/CXCL12) axis has also emerged as an important target for genome-edited stem cell therapies. Using TALEN-mediated genome editing, Zhang et al. generated SDF-1 knock-in human amniotic mesenchymal stem cells and demonstrated significantly enhanced angiogenesis, capillary density, blood perfusion recovery, and limb salvage in a murine hindlimb ischemia model [23]. Because SDF-1 is a critical chemokine that regulates progenitor cell recruitment, angiogenesis, and tissue repair through the CXCR4 signaling pathway.

In addition to directly promoting angiogenesis, genome editing has been employed to enhance adaptation to ischemic microenvironments. Pan et al. proposed CRISPR/dCas9-based gene regulation as a platform for improving stem cell survival under ischemic conditions by modulating stress-response and survival pathways [29]. Similarly, Yan et al. generated FOXO3-engineered human embryonic stem cell-derived vascular cells using CRISPR/Cas9-mediated editing [28]. Activation of the FOXO3 longevity-associated allele improved resistance to oxidative stress, delayed cellular senescence, and enhanced vascular regenerative capacity following transplantation into ischemic tissues [28]. These findings suggest that genome editing can enhance not only angiogenesis but also the long-term persistence and functionality of transplanted cells.

Several studies have further demonstrated that genome editing can augment the secretion of therapeutic cytokines and growth factors. Chang et al. generated inducible hepatocyte growth factor (HGF)-secreting mesenchymal stem cells using TALEN-mediated genome editing. The engineered cells exhibited enhanced angiogenic activity and improved vascular regeneration through sustained HGF release [24]. Likewise, Meng et al. developed interleukin-10 (IL-10)-secreting mesenchymal stem cells via TALEN-mediated gene editing and demonstrated attenuation of post-infarction inflammation and left ventricular remodeling following myocardial infarction [26]. These reports suggest that genome editing can enhance anti-inflammatory and immunomodulatory paracrine effects as well as pro-angiogenic activity.

### 4.3. Promoting Endothelial Maturation and Vascular Stability

Generating endothelial-like cells is only the first step toward successful vascular regeneration. Equally important is the development of mature and stable vascular networks capable of sustaining long-term tissue perfusion [30]. A recurring limitation of stem cell-derived endothelial cells is their tendency to exhibit immature phenotypes [31]. Deficiencies in barrier integrity, junctional organization, and vascular stability can compromise therapeutic outcomes and contribute to regression of newly formed vessels after transplantation [32]. These challenges have prompted growing interest in strategies that promote endothelial maturation rather than focusing solely on endothelial differentiation [31].

Genome editing has also been utilized to investigate endothelial maturation and functional vascular phenotypes beyond angiogenesis [30,31]. For example, CRISPR/Cas9-mediated introduction of the TIE2^L914F^ mutation into iPSC-derived endothelial cells generated a disease-relevant model of venous malformations [33]. The resulting endothelial cells showed abnormal vascular signaling, increased migration, and reduced responses to shear stress. These findings highlight the role of mechanotransduction pathways in endothelial maturation, vascular remodeling, and long-term vessel stability.

From a clinical perspective, durable vascular regeneration requires a balance between vessel formation and vessel stabilization [32,34]. Excessive emphasis on angiogenic stimulation alone may produce immature and poorly organized vascular networks. In contrast, strategies that combine enhanced differentiation, improved ischemic tolerance, optimized paracrine signaling, and endothelial maturation are more likely to generate durable therapeutic outcomes [30,31,32,34].

## 5. Therapeutic Applications of Gene-Edited Stem Cells in Ischemic Vascular Diseases

**Figure 3:**
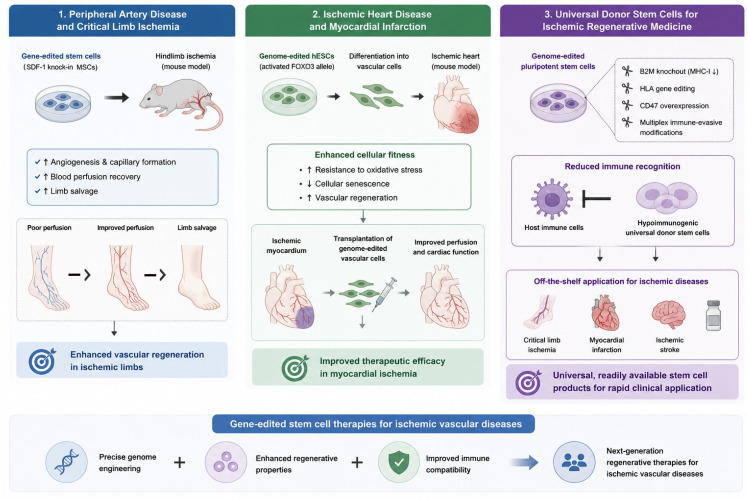
Therapeutic applications of gene-edited stem cells for ischemic vascular diseases. Schematic overview of the major therapeutic applications of genome-edited stem cells in ischemic vascular diseases. In peripheral artery disease (PAD) and critical limb ischemia (CLI), gene-edited stromal cell-derived factor-1α (SDF-1α) knock-in stem cells enhance angiogenesis, capillary formation, blood perfusion recovery, and limb salvage. In ischemic heart disease and myocardial infarction, genome-edited stem cell-derived vascular cells carrying an activated FOXO3 allele exhibit increased resistance to oxidative stress, delayed cellular senescence, and improved vascular regeneration, resulting in enhanced cardiac repair and functional recovery. Genome-editing technologies have also enabled the generation of hypoimmunogenic universal donor stem cells through targeted modification of immune-recognition pathways, including β2-microglobulin (B2M) disruption, HLA-related gene editing, and CD47 overexpression. These immune-evasive stem cells demonstrate reduced immune rejection and prolonged graft survival, supporting the development of off-the-shelf regenerative therapies for ischemic disorders such as critical limb ischemia, myocardial infarction, and ischemic stroke. Collectively, the integration of precise genome engineering, enhanced regenerative function, and improved immune compatibility provides a promising platform for next-generation stem cell therapies for ischemic vascular diseases. Panel colors distinguish different disease applications, while arrows and checkmarks indicate mechanistic progression and major therapeutic benefits, respectively. The figure was generated using OpenAI ChatGPT (GPT-5.5) image generation tools and subsequently reviewed and edited by the authors.

The integration of genome-editing technologies with stem cell engineering has created new opportunities for regenerative therapies targeting ischemic vascular diseases. Traditional stem cell therapies have demonstrated promising angiogenic and reparative effects; however, inconsistent cell survival, limited engraftment, and immune rejection remain major barriers to clinical translation. Recent advances in CRISPR/Cas9- and TALEN-based genome editing have enabled the generation of stem cells with enhanced regenerative properties and improved transplantation compatibility, providing a foundation for next-generation cell therapies for ischemic disorders [28]. An overview of the therapeutic applications of gene-edited stem cells in ischemic vascular diseases is presented in Figure 3.

### 5.1. Peripheral Artery Disease and Critical Limb Ischemia

Peripheral artery disease (PAD) and critical limb ischemia (CLI) represent attractive targets for gene-edited stem cell therapy because therapeutic efficacy can be readily evaluated through blood flow recovery, neovascularization, and limb salvage. A representative example was reported by Zhang et al., who generated TALEN-mediated SDF-1 knock-in human amniotic mesenchymal stem cells and demonstrated significantly enhanced angiogenesis, capillary formation, and perfusion recovery in a murine hindlimb ischemia model [23]. These findings provided direct evidence that precise genome engineering can improve the vascular regenerative capacity of stem cells under ischemic conditions. As genome-editing technologies continue to evolve, we anticipate that combining pro-regenerative genetic modifications with immune-evasive engineering strategies will be critical for the development of next-generation stem cell therapies for ischemic vascular diseases.

### 5.2. Ischemic Heart Disease and Myocardial Infarction

The ischemic myocardium presents an extremely hostile microenvironment characterized by hypoxia, oxidative stress, and inflammation, which substantially limits the survival of transplanted cells. Genome editing has therefore been explored as a strategy to enhance the functional resilience of stem cell-derived vascular cells. In a notable study, Yan et al. introduced an activated FOXO3 allele into human embryonic stem cells through precise genome editing and subsequently differentiated them into vascular cells [28]. The engineered vascular cells exhibited increased resistance to oxidative injury, delayed cellular aging, and enhanced vascular regeneration in a mouse ischemia model, suggesting that targeted genetic modification can improve both cellular fitness and therapeutic efficacy following transplantation. Together, these data point to genome-edited vascular cells as a promising future platform for treating myocardial ischemia and ischemic cardiomyopathy. Although clinical application remains in the early stages, advances in genome engineering may facilitate the development of more durable and functionally enhanced cardiovascular cell products.

### 5.3. Universal Donor Stem Cells for Ischemic Regenerative Medicine

One of the major translational barriers to stem cell-based regenerative therapies is immune-mediated rejection following allogeneic transplantation. To overcome this challenge, genome-editing technologies have been employed to generate hypoimmunogenic stem cells with reduced susceptibility to host immune recognition. Early studies demonstrated that targeted disruption of β2-microglobulin (B2M), a key component of major histocompatibility complex class I (MHC-I) antigen presentation, significantly reduced the immunogenicity of human embryonic stem cells and improved their compatibility for allogeneic transplantation [35]. Building upon these findings, Deuse et al. generated universal donor-like human pluripotent stem cells through modification of immune-recognition pathways combined with CD47-mediated immune-evasion mechanisms, resulting in markedly prolonged graft survival after transplantation [21]. Similarly, Han et al. demonstrated that multiplex CRISPR/Cas9-mediated editing of HLA-associated genes effectively reduced immune recognition and enhanced transplantation compatibility [27]. Although these approaches have not been specifically developed for ischemic vascular disease models, they have important translational implications for regenerative vascular medicine. In clinical settings requiring rapid intervention, such as critical limb ischemia, myocardial infarction, or ischemic stroke, standardized off-the-shelf universal donor stem cell products could reduce manufacturing time, improve scalability, lower production costs, and increase treatment accessibility. Therefore, despite remaining largely at the preclinical stage, hypoimmunogenic genome-edited stem cells represent one of the most promising applications of genome editing for the future clinical translation of regenerative therapies for ischemic vascular diseases.

### 5.4. Future Clinical Translation

Despite encouraging preclinical results, the clinical translation of gene-edited stem cell therapies remains at an early stage. Future development will require optimization of editing efficiency, comprehensive evaluation of long-term genomic stability, and rigorous assessment of safety. In addition, scalable manufacturing platforms and regulatory frameworks must be established before widespread clinical implementation becomes feasible. Nevertheless, accumulating evidence suggests that genome editing can substantially enhance the regenerative potential of stem cells through improved angiogenesis, increased resistance to ischemic stress, and reduced immune rejection. These advances may ultimately enable the development of effective and widely accessible regenerative therapies for ischemic vascular diseases.

## 6. Safety and Ethical Considerations

### 6.1. Genome-Editing Delivery Platforms and Translational Challenges

The therapeutic success of genome-edited stem cells depends not only on editing accuracy but also on the efficient and safe delivery of genome-editing components into target cells. Various delivery platforms have been developed for stem cell engineering, including viral vectors, electroporation-based approaches, and non-viral delivery systems, each presenting distinct advantages and limitations [36].

Viral vectors, particularly lentiviral vectors and adeno-associated viruses (AAVs), remain widely used because of their high delivery efficiency and robust transgene expression [36]. Lentiviral vectors are highly effective for ex vivo stem cell modification but may raise concerns regarding insertional mutagenesis due to genomic integration. In contrast, AAV vectors generally exhibit favorable safety profiles and lower immunogenicity; however, their limited packaging capacity can restrict the delivery of large genome-editing cargos such as base editors and prime editors [37].

Electroporation-mediated delivery of CRISPR/Cas9 ribonucleoprotein (RNP) complexes has emerged as an attractive alternative for stem cell engineering. Because RNPs are delivered directly into cells without vector integration, genome-editing activity is transient, reducing the risk of prolonged nuclease expression and off-target effects [38]. This strategy has demonstrated high editing efficiency in multiple stem cell populations and is increasingly considered suitable for clinical-scale ex vivo genome editing [39].

Non-viral delivery platforms, including lipid nanoparticles, polymeric carriers, and engineered extracellular vesicles, have also attracted considerable interest because they offer improved safety and manufacturing flexibility compared with viral systems [39]. Nevertheless, challenges related to intracellular delivery efficiency, tissue-specific targeting, and large-scale production remain significant barriers to clinical translation [37].

For stem cell-based therapies targeting ischemic vascular diseases, delivery strategies must not only achieve efficient genome editing but also preserve stem cell viability, regenerative capacity, and therapeutic potency. Therefore, continued advances in delivery technologies will be essential for the safe and effective clinical translation of genome-edited stem cell therapies [39].

### 6.2. Genomic Safety, Quality Control, and Ethical Considerations

One of the most frequently discussed challenges is off-target genome modification. Even highly optimized editing systems may occasionally interact with unintended genomic sites, potentially leading to unwanted mutations, chromosomal abnormalities, or alterations in cellular behavior [40]. Such events are particularly concerning in stem cell-based therapies because edited cells may persist and proliferate for extended periods after transplantation. Although improvements in guide RNA design, high-fidelity Cas variants, and transient delivery approaches have substantially reduced off-target activity, complete elimination of risk has not yet been achieved [9]. Consequently, comprehensive genomic characterization remains an essential component of preclinical development.

Another major concern relates to genomic stability [41]. Stem cells undergo extensive expansion during manufacturing, and prolonged culture itself can introduce genetic and epigenetic alterations. When genome editing is combined with large-scale cell expansion, distinguishing naturally acquired abnormalities from editing-induced changes becomes increasingly challenging [41]. Even subtle genomic alterations may influence differentiation potential, cellular fitness, or long-term safety. For this reason, regulatory agencies are placing growing emphasis on genomic surveillance and quality-control testing throughout the manufacturing process.

Tumorigenicity represents an additional obstacle, particularly in therapies involving pluripotent stem cells [42]. Both embryonic stem cells and induced pluripotent stem cells possess extensive proliferative capacity, which contributes to their regenerative potential but also raises concerns regarding uncontrolled growth and teratoma formation [42]. Although differentiation protocols continue to improve, complete elimination of residual undifferentiated cells remains difficult. Safety mechanisms such as inducible suicide switches and drug-responsive elimination systems have therefore attracted considerable interest as supplementary safeguards. Even so, their long-term reliability under clinical conditions requires further validation.

Beyond these biological concerns, robust quality-control strategies are essential for the safe clinical translation of genome-edited stem cell products. Comprehensive genomic characterization should extend beyond assessment of editing efficiency and include cytogenetic analyses such as karyotyping and chromosomal integrity testing to identify large-scale genomic abnormalities [43]. In addition, whole-genome sequencing (WGS) and targeted off-target profiling technologies can be employed to detect unintended genomic alterations and evaluate editing precision. For example, WGS analyses of CRISPR/Cas9- and TALEN-edited human stem cell clones revealed a low incidence of off-target mutations, supporting the feasibility of genome editing while underscoring the need for rigorous genomic characterization prior to clinical translation [44]. Together, these approaches provide critical information regarding the genetic integrity of edited cell populations and help minimize the risk of introducing harmful mutations into clinical products.

Long-term culture monitoring represents another important component of quality assurance. Because stem cells often undergo extensive expansion during manufacturing, continuous surveillance is required to identify genomic instability, clonal selection, or phenotypic drift that may emerge during prolonged culture [45]. Furthermore, residual undifferentiated pluripotent stem cells should be carefully assessed using molecular and phenotypic detection methods to reduce the risk of teratoma formation after transplantation. Functional potency assays evaluating angiogenic activity, immunomodulatory properties, or resistance to ischemic stress may also be incorporated to confirm the intended therapeutic function of edited cells. Together, these quality-control measures may serve as essential release criteria for clinical-grade manufacturing and contribute to the safety, consistency, and reproducibility of genome-edited stem cell therapies [46].

Immune-related issues introduce another layer of complexity. Allogeneic stem cell products may provoke both innate and adaptive immune responses, reducing therapeutic efficacy and limiting long-term engraftment [21]. Genome-editing strategies designed to reduce immunogenicity have shown promising results in preclinical studies; however, immune evasion itself raises new questions. Cells that become less visible to immune surveillance may theoretically acquire a survival advantage that complicates the detection and elimination of abnormal cell populations [47]. Balancing immune compatibility with biological safety therefore remains a delicate challenge.

Overall, these considerations suggest that successful clinical translation will depend on more than technological innovation alone. Robust regulatory oversight, long-term patient monitoring, transparent risk assessment, and meaningful public engagement will be equally important.

## 7. Future Perspectives

### 7.1. Next-Generation Genome-Editing Technologies

Genome-editing technologies continue to evolve rapidly beyond conventional CRISPR/Cas9 systems. Among the most significant advances are base editing and prime editing, which enable precise genetic modifications without introducing double-strand DNA breaks [10]. By reducing reliance on error-prone DNA repair pathways, these platforms offer improved precision and broaden the range of potentially treatable genetic targets [13].

For regenerative medicine, future applications are likely to extend beyond correction of individual genes. Because ischemic vascular diseases involve complex interactions among angiogenic, inflammatory, and metabolic pathways, multiplex editing strategies capable of regulating multiple biological networks simultaneously may become increasingly important [48]. In parallel, CRISPR-based transcriptional and epigenetic editors provide an alternative approach by modulating gene expression without permanently altering DNA sequences, potentially improving safety while maintaining therapeutic efficacy [49].

Further improvements in editing specificity are expected through the development of high-fidelity editors, optimized guide RNAs, and advanced computational prediction tools [50]. Artificial intelligence is also emerging as a valuable resource for identifying gene networks involved in vascular regeneration and for designing more effective engineering strategies [39]. Nevertheless, challenges related to editing efficiency, delivery, manufacturing complexity, and large-scale production remain unresolved.

Although base editing and prime editing have emerged as promising next-generation genome-editing technologies, their application in stem cell-based therapies for ischemic vascular diseases remains limited. To date, most studies utilizing these approaches have focused on monogenic disorders, hematologic diseases, and proof-of-concept cellular models rather than vascular regenerative applications. Nevertheless, because these technologies can introduce precise nucleotide modifications without generating double-strand DNA breaks, they may offer important advantages over conventional nuclease-based editing systems, including improved editing accuracy and potentially enhanced safety profiles. Future studies should investigate whether base editing and prime editing can be applied to engineer stem cells with enhanced angiogenic capacity, ischemia resistance, immunomodulatory function, and vascular regenerative potential.

Overall, next-generation genome-editing technologies are shifting the field from simple gene correction toward comprehensive cellular programming, creating new opportunities for the development of advanced regenerative therapies.

### 7.2. Expanded Clinical Application and Translation

The next decade is expected to be a pivotal period for the clinical translation of gene-edited stem cell therapies. Growing evidence from preclinical studies, combined with recent successes in genome editing-based medicine, has strengthened confidence in the therapeutic potential of engineered cell products [51].

Several factors are likely to drive future clinical adoption. The continued global burden of ischemic vascular diseases creates a strong unmet medical need, while advances in automated manufacturing, scalable differentiation protocols, and quality-control systems are gradually improving the feasibility of large-scale cell production [52]. In addition, hypoimmunogenic and universal donor cell platforms may facilitate the transition from personalized therapies to off-the-shelf regenerative products [21].

Despite these advances, successful translation will require continued attention to safety, regulatory oversight, manufacturing consistency, and economic accessibility [53]. Ultimately, the convergence of genome editing, stem cell biology, biomaterials science, artificial intelligence, and advanced biomanufacturing may enable regenerative therapies that restore vascular function and promote durable tissue repair. Although significant challenges remain, gene-edited stem cell therapy is increasingly emerging as a realistic therapeutic strategy for ischemic vascular diseases. In our view, early clinical translation will most likely occur in indications where robust preclinical models and clear surrogate endpoints for vascular regeneration are already available.

However, it is important to recognize that the majority of studies discussed in this review remain at the preclinical stage, with evidence derived primarily from in vitro experiments and animal models of myocardial infarction and hindlimb ischemia. To date, no gene-edited stem cell therapy has been approved for the treatment of ischemic vascular diseases, and clinical experience remains extremely limited. Therefore, although preclinical findings have demonstrated considerable therapeutic potential, further studies are required to establish long-term safety, manufacturing feasibility, regulatory compliance, and clinical efficacy before routine clinical application can be realized.

## 8. Conclusions

Gene editing has rapidly evolved into a transformative platform for engineering stem cells with enhanced regenerative capacity, improved stress resistance, and reduced immunogenicity. In the context of ischemic vascular disease, these technologies offer a powerful means to address several of the principal barriers that have limited the clinical success of conventional stem cell therapies, including poor survival, insufficient engraftment, functional heterogeneity, and immune rejection. The convergence of CRISPR/Cas9, base editing, prime editing, and related precision-engineering approaches with stem cell biology has therefore opened a new avenue for the development of next-generation regenerative therapies [39].

Beyond simple genetic correction, gene editing now enables more sophisticated strategies aimed at improving angiogenesis, strengthening paracrine signaling, promoting endothelial maturation, and generating hypoimmunogenic or universal donor cell products [21,54]. These advances suggest that the most effective future cell therapies will likely be those designed not only to replace damaged cells but also to reshape the ischemic microenvironment and sustain long-term vascular repair [54]. In particular, the integration of pro-regenerative and immune-evasive modifications may provide a more durable and clinically practical framework for treating peripheral artery disease, myocardial infarction, and other ischemic vascular disorders.

Despite these promising developments, important challenges remain before widespread clinical translation can be achieved. Editing safety, genomic stability, delivery efficiency, scalable manufacturing, and regulatory oversight must all be addressed through rigorous preclinical testing and standardized quality-control strategies [39,55]. Nevertheless, continued progress in genome engineering, stem cell manufacturing, and translational cell biology is likely to accelerate the emergence of effective, off-the-shelf regenerative products. Taken together, gene-edited stem cell therapies hold considerable promise as a realistic and potentially durable treatment strategy for ischemic vascular diseases.

## Figures and Tables

**Table 1:** Representative genome-editing strategies evaluated in ischemic vascular disease models and related regenerative applications.

Stem Cell Type	Genome-Editing Strategy	Target Gene(s)	Primary Function	Disease Model	Key Findings	Evidence Type	Study
UCB-MSCs	TALEN-mediated knock-in	HGF	Angiogenesis	Hindlimb ischemia	Enhanced HGF secretion and improved angiogenesis	Direct Ischemic Evidence	[24]
UCB-MSCs	TALEN-mediated targeted integration	VEGF	Angiogenesis and tissue repair	Myocardial infarction	Improved cardiac function, reduced fibrosis, increased vascularization	Direct Ischemic Evidence	[25]
Human amniotic MSCs	TALEN-mediated knock-in	IL-10	Anti-inflammatory activity	Myocardial infarction	Reduced inflammation and attenuated ventricular remodeling	Direct Ischemic Evidence	[26]
Human amniotic MSCs	TALEN-mediated knock-in	SDF-1/CXCL12	Angiogenesis and stem cell homing	Hindlimb ischemia	Improved perfusion recovery, angiogenesis, and limb salvage	Direct Ischemic Evidence	[23]
hiPSCs	CRISPR/Cas9 editing	MHC-I pathway, CD47	Immune evasion	Transplantation model	Prolonged graft survival and immune tolerance	Supportive/Translational	[21]
hPSCs	Multiplex CRISPR/Cas9 editing and targeted knock-in	HLA-A, HLA-B, HLA-C, CIITA, PD-L1, HLA-G, CD47	Immune evasion	Transplantation model	Reduced immune recognition and improved compatibility	Supportive/Translational	[27]
hESC-derived vascular cells	CRISPR/Cas9 editing	FOXO3	Oxidative stress resistance	Ischemic vascular injury model	Enhanced vascular regeneration and delayed senescence	Direct Ischemic Evidence	[28]

## Data Availability

No new data were created or analyzed in this study.
